# Fe-flavonoid nanozyme as dual modulator of oxidative stress and autophagy for acute kidney injury repair

**DOI:** 10.7150/thno.111874

**Published:** 2025-07-28

**Authors:** Ranran Luo, Zhongsheng Xu, Chenxi Zhang, Zening Zhang, Pengchen Ren, Xiaojing He, Jingjing Zhang, Yun Liu

**Affiliations:** 1 Department of Radiology, the Second Affiliated Hospital of Chongqing Medical University, Chongqing, 400010, P. R. China.; 2 Departments of Diagnostic Radiology, Surgery, Chemical and Biomolecular Engineering, and Biomedical Engineering, Yong Loo Lin School of Medicine and College of Design and Engineering, National University of Singapore, Singapore 119074, Singapore.; 3 Clinical Imaging Research Centre, Centre for Translational Medicine, Yong Loo Lin School of Medicine, National University of Singapore, Singapore 117599, Singapore.; 4 Nanomedicine Translational Research Program, Yong Loo Lin School of Medicine, National University of Singapore, Singapore 117597, Singapore.; 5 Theranostics Center of Excellence (TCE), Yong Loo Lin School of Medicine, National University of Singapore, 11 Biopolis Way, Helios, Singapore 138667, Singapore.

**Keywords:** natural flavonoids, chelator, acute kidney injury, oxidative stress, autophagy

## Abstract

**Background:** Acute kidney injury (AKI), marked by a high mortality rate, remains a significant clinical challenge owing to limited therapeutic options. Oxidative stress is a key driver of AKI pathogenesis, underscoring the urgent need for innovative interventions. Recent advances demonstrate the potential of reshaping the oxidative stress microenvironment and activating intracellular autophagy to facilitate tissue repair. Nanotechnology-based antioxidants are emerging as promising approaches for AKI. Here, we present a novel nanoscale natural antioxidant platform for AKI treatment, incorporating reactive oxygen species (ROS) scavenging, oxidative stress modulation, anti-inflammatory properties and autophagy activation, which leverages these synergistic functions and lays the groundwork for clinical translation of next-generation nanotherapeutics in AKI.

**Methods:** We synthesized a Fe-flavonoid nanozyme (FD@BSA) composed of ferric chloride hexahydrate, dihydromyricetin (DMY), and bovine serum albumin (BSA). FD@BSA integrated DMY's antioxidant and autophagy-activating functions with iron-mediated catalytic activity. Its therapeutic efficacy was evaluated in two oxidative stress-driven renal injury models: H_2_O_2_-induced ROS overload in human renal proximal tubular epithelial (HK-2) cells and glycerol-mediated AKI mice. Mechanistic studies employed laser confocal microscopy to visualize intracellular ROS scavenging and autophagy activation, while Western blotting and immunohistochemistry assessed protein expression and tissue-level pathology.

**Results:** After intravenous administration, FD@BSA nanozyme selectively accumulated in the kidneys of water-restricted, glycerol-induced AKI mice. *In vitro* studies demonstrated that FD@BSA significantly decreased ROS accumulation in HK-2 cells, enhanced cell viability, attenuated inflammatory responses, and induced mitophagy, thereby preserving cellular homeostasis and alleviating injury. *In vivo*, FD@BSA treatment markedly ameliorated glycerol-induced AKI. Mechanistically, this protective effect was mediated by inhibition of NOD-like receptor family pyrin domain containing 3 (NLRP3) inflammasome activation and upregulation of light chain 3 (LC3)-dependent autophagy, which together reduced ROS-driven cellular damage and mitigated renal injury, highlighting FD@BSA as a promising strategy for AKI.

**Conclusion:** This study establishes FD@BSA nanozyme as a versatile nanotherapeutic platform for AKI, which can effectively remodel the oxidative stress microenvironment by scavenging excessive ROS and activating intracellular autophagy. Such multifunctionality extends FD@BSA's applicability beyond AKI to other ROS-driven pathologies, positioning it as a next-generation, nanotechnology-based strategy for the treatment of oxidative stress-related diseases.

## Introduction

Acute kidney injury (AKI) is a common and severe complication in critically ill patients defined by inflammatory tubular damage and a potentially reversible decline in renal function 1, 2. Epidemiological studies indicate that AKI occurs in 10-15% of hospitalized patients and up to 50% of intensive care unit admissions, underscoring its global health impact 3, 4. Clinically, AKI manifests as an abrupt reduction in glomerular filtration rate accompanied by elevated blood urea nitrogen and serum creatinine levels 5. Current management is limited to minimizing nephrotoxic insults, providing supportive care, and preventing secondary complications 6-8. However, effective noninvasive therapies that actively promote renal repair remain unavailable, and renal replacement therapy serves only as a temporizing intervention.

Oxidative stress, driven by excessive reactive oxygen species (ROS) generation, is a key mediator of AKI pathogenesis 9. ROS overproduction damages lipids, proteins, and DNA, precipitating acute tubular injury and renal dysfunction 10, 11. When ROS generation outpaces antioxidant defenses, apoptosis, necrosis, and inflammatory cascades are unleashed, exacerbating tissue damage 12, 13. Current strategies aim to scavenge ROS and reactive nitrogen species (RNS) to restore redox homeostasis 14-16. In addition, ROS overload induces nuclear and mitochondrial injury, activates the NLRP3 inflammasome, and triggers apoptotic signaling. Studies indicate that ROS neutralization attenuates oxidative stress, dampens inflammation, and inhibits cell death in contrast-induced AKI models 17-19. Nevertheless, ROS scavengers alone are typically insufficient for rapid and complete restoration of cellular homeostasis.

Autophagy, a tightly regulated catabolic process essential for cellular homeostasis, has emerged as a potential therapeutic target in AKI 20-22. It mediates the removal of damaged organelles and oxidized biomolecules, maintaining intracellular equilibrium 23, 24. Basal autophagy occurs at low levels in all cell types and can be activated *via* Sirtuin 1 (Sirt1), phosphoinositide 3-kinase (PI3K), and mitogen-activated protein kinase (MAPK) pathways 25, 26. Increased autophagy promotes the degradation of oxidized proteins and clearance of ROS-generating organelles, such as mitochondria and peroxisomes, thereby mitigating oxidative stress and preventing ROS-induced cellular damage 27, 28. Despite its promise, few clinical strategies have harnessed both ROS scavenging and autophagy induction for AKI therapy.

Natural flavonoids such as dihydromyricetin (DMY), quercetin, kaempferol, and naringenin exert diverse physiological and biochemical effects 29, 30. Among them, DMY displays potent antioxidant and anti-inflammatory activities that mitigate neurological and cardiovascular disorders and other free radical-related inflammatory conditions 31, 32. Notably, DMY induces autophagy *via* the Sirt1-LC3 pathway, thereby preserving cellular homeostasis 33, 34. However, chemical instability, poor aqueous solubility, and limited membrane permeability severely constrain the bioavailability of these flavonoids 35, 36. To address these limitations, we previously demonstrated that Fe^3+^ self-assembles with natural compounds through chelation with -OH groups to form nanoparticles, substantially enhancing solubility, safety, and bioavailability 37.

**Scheme 1:**
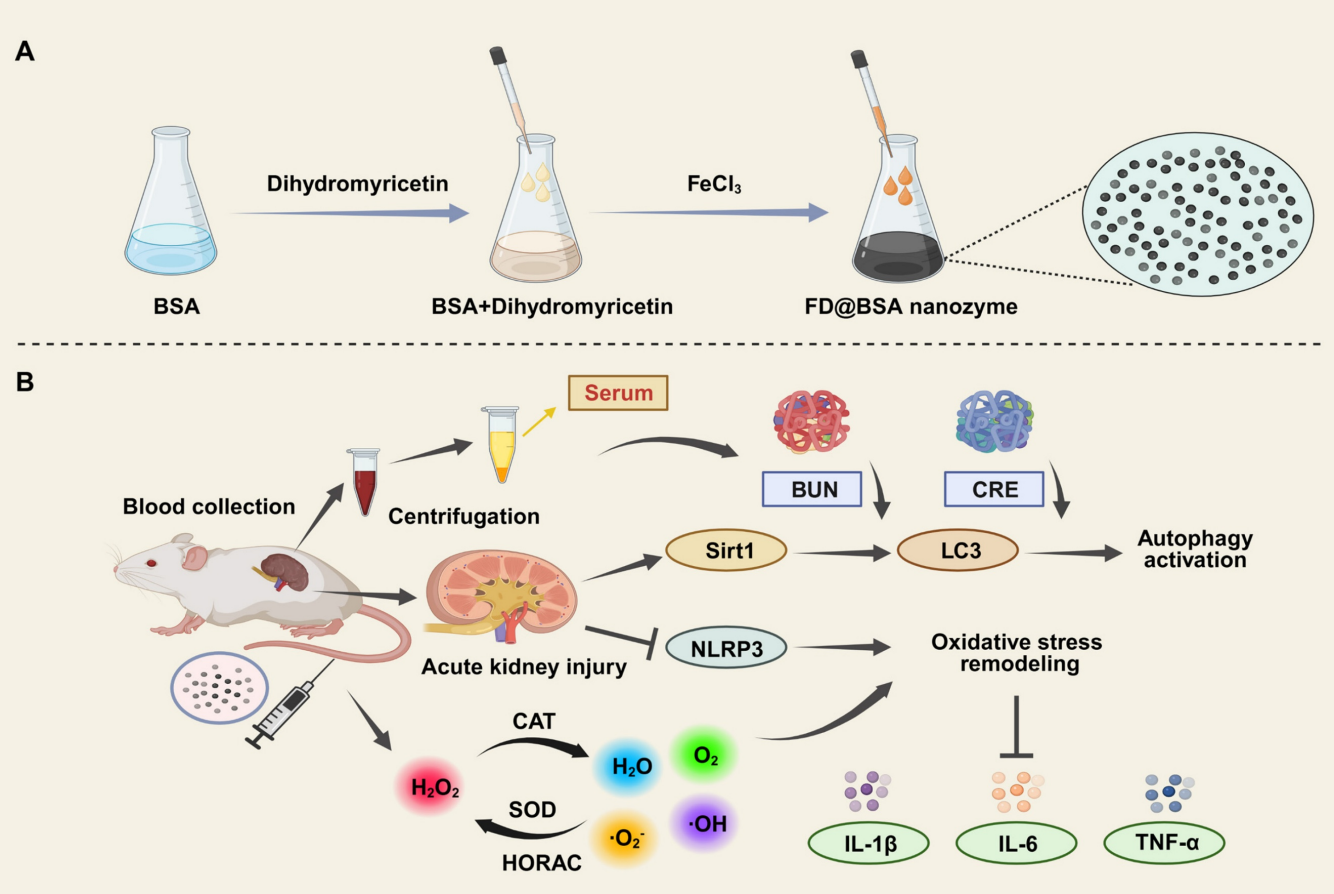
(A) Schematic illustration of FD@BSA synthesis. (B) FD@BSA-mediated remodeling of the oxidative stress microenvironment and activation of autophagy during AKI therapeutic repair.

In this study, we developed an ultrasmall Fe^3+^-coordinated DMY nanozyme (FD@BSA) *via* a facile coordination self-assembly strategy. Bovine serum albumin (BSA) served as a biocompatible, water-soluble scaffold onto which DMY was adsorbed and subsequently chelated with Fe^3^⁺ to form FD@BSA (Scheme 1). FD@BSA remodeled the oxidative stress microenvironment by scavenging ROS, inhibiting apoptosis, and activating autophagy 38-40. Mechanistically, FD@BSA dose-dependently suppressed NLRP3 inflammasome activation, evidenced by reduced apoptosis-associated speck-like protein containing a CARD (ASC) speck formation, diminished Caspase-1 cleavage, and lower inflammatory cytokine release. Concurrently, it upregulated Sirt1 and LC3 expression and promoted autophagosome biogenesis in inflammatory cells. Additionally, FD@BSA increased the anti‑apoptotic protein B-cell lymphoma-2 (Bcl-2) while decreasing pro-apoptotic protein Bcl-2 associated X protein (Bax) expression. These findings highlight a multifunctional nanozyme strategy to correct oxidative stress and autophagy dysregulation, positioning FD@BSA as a promising nanoplatform for AKI and other ROS-driven diseases.

## Materials and Methods

### Materials

BSA (P1628334) and DMY (P2107068) were obtained from Admas-beta (Shanghai, China). Iron(III) chloride hexahydrate (G10101B) was purchased from General Reagent (Shanghai, China). Minimum essential medium (MEM; PM150410) and Dulbecco's modified Eagle's medium (DMEM; PM150210) were purchased from Pricella (Wuhan, China). Paraformaldehyde (P0099), penicillin-streptomycin (C0222), trypsin (C0201), DAPI staining reagent (C1002), Cell Counting Kit-8 (CCK-8) (C0037), JC-1 dye (C2006), calcein/propidium iodide staining and sodium dodecyl sulfate-polyacrylamide gel electrophoresis (SDS-PAGE) kit (P0012A) were purchased from Beyotime (Shanghai, China). N-acetylcysteine (NAC) was obtained from Solarbio (Beijing, China). Ultrafiltration centrifuge tubes (UFC910008) were purchased from Merck KGaA (Darmstadt, Germany). Sulfo-cyanine 5 (Cy5) was acquired from MCE (New Jersey, USA). Fetal bovine serum (FBS; 10100147) was obtained from Gibco (Thermo Fisher Scientific, Shanghai, China). Annexin V-FITC/PI apoptosis kit (E-CK-A211) was purchased from Elabscience (Wuhan, China). Calcein-AM/PI double staining kit (C542), mitophagy detection kit (MD01) and ROS assay kit (AD10) were purchased from Dojindo Laboratories (Kumamoto, Japan). NLRP3 (D4D8T), ASC/TMS1 (E1E3I) and GAPDH (#5174) were obtained from Cell Signaling Technology (Beverly, MA, USA). Anti-LC3B (ab48394) and anti-pro Caspase-1 (ab179515) antibodies were obtained from Abcam (Cambridge, MA, USA). Enzyme-linked immunosorbent assay (ELISA) kits, including human IL-6 (EK0410), TNF-α (EK0525), and IL-1β (EK0392) kits, were acquired from Boster (Wuhan, China), while mouse IL-6 (AF2163-A), TNF-α (AF2132-A), and IL-1β (AF2040-A) kits were purchased from Aifang (Hunan, China).

### Preparation of FD@BSA nanozyme

Colloidal FD@BSA nanoparticles were synthesized according to our previous study 37. Typically, 1 mL of 50 mg/mL DMY solution (DMSO) was added into an aqueous solution of BSA (10 mL, 1 mg/mL) with stirring for 1 h, followed by the addition of an aqueous solution of FeCl_3_•6H_2_O (1 mL, 10 mg/mL) for another 12 h. The FD@BSA nanozyme was isolated by ultrafiltration centrifugation (MWCO 100 kDa) three times, and the product supernatant was collected. Cy5-labeled nanozyme was fabricated using the same method, in which BSA was labeled with Cy5.

### Characterization of FD@BSA nanozyme

FD@BSA nanozyme was observed by transmission electron microscopy (TEM; HT7700, Hitachi, Japan). The hydrodynamic diameters and zeta potentials of FD@BSA were analyzed by a surface zeta potential and particle size analyzer at room temperature (Zetasizer, Malvern, Britain). X-ray photoelectron spectroscopy (XPS) was carried out with an ESCALAB 250Xi spectrometer (Thermo Fisher Scientific) equipped with a monochromatic Al Kα X-ray source. Fourier transform infrared (FT-IR) spectroscopy (Nicolet iS5, Thermo Fisher Scientific, USA) was performed to acquire the infrared absorption of BSA and DMY in FD@BSA. The formula used to calculate the relevant enzyme activity was as follows: enzyme activity (U/mg) = *I* × V/(50% × *v* × C), where *I* represents the inhibition rate (%), V represents the total volume of the reaction system (mL), *v* represents the actual volume added (mL), and C represents the concentration (mg/mL).

### ROS/RNS scavenging properties of FD@BSA nanozyme *in vitro*

Superoxide anion (•O_2_^-^) was generated using the xanthine/xanthine oxidase system and monitored *via* reduction of the tetrazolium salt 2-(2-methoxy-4-nitrophenyl)-3-(4-nitrophenyl)-5-(2,4-dinitrophenyl)-2H-tetrazolium (WST-8) and electron spin resonance (ESR) spectroscopy was employed to quantify the scavenging capacity of FD@BSA (25, 50, 125, 250 and 500 μg/mL) against •O_2_^-^.

Hydroxyl radical (•OH) scavenging activity was measured using the hydroxyl radical antioxidant capacity (HORAC) assay, based on •OH-induced oxidation of methylene blue as well as generation of •OH by FeSO_4_/H_2_O_2_ system (665 nm irradiation), and the radical scavenging was detected by ESR spectroscopy with FD@BSA.

Different concentrations of FD@BSA were added to 6 mL of PBS (pH 7.4) and 200 μL of 30% H_2_O_2_, and the changes of oxygen concentration in the solution were monitored by a portable dissolved oxygen meter for 15 min.

0.1 mM ethanolic solution of 1,1-diphenyl-2-picrylhydrazyl (DPPH) was mixed with different concentrations of FD@BSA. After incubation in the dark for 30 min at 25 ℃, scavenging activity against the nitrogen-centered radical was quantified by measuring absorbance at 517 nm.

### Cell culture

HK-2 cells and Raw 264.7 cells obtained from Pricella (Wuhan, China) were cultured in MEM and DMEM supplemented with 10% FBS, 100 U/mL penicillin and 100 mg/mL streptomycin in a humidified incubator containing 5% CO_2_ maintained at 37 ℃.

### Toxicological evaluation

The cytotoxicity of FD@BSA nanozyme was evaluated in HK-2 cells, Raw 264.7 cells and erythrocytes. HK-2 cells and Raw 264.7 cells were seeded into a 96-well plate at a density of 5 × 10^3^ cells per well. Cell survival upon exposure to increasing concentrations of FD@BSA (50, 100, 200 and 400 μg/mL), on the basis of the weight of nanoparticles (NPs) were determined by a CCK-8 assay after 24 h. A hemolytic experiment was conducted as recently described. Briefly, 300 μL of mouse erythrocytes suspended in 0.9% NaCl solution were incubated with FD@BSA (50, 100, 200, 400 and 800 μg/mL) at 37 °C for 2 h.

### Cell experiments

HK-2 cells were plated in 12-well plates at a density of 1.5 × 10^5^ cells per well and separately cultured in confocal dishes overnight. After adhering, the cells were exposed to Cy5-labeled FD@BSA (50 μg/mL) and H_2_O_2_ (20 μM) at 37 °C for 6 h. Nanozyme endocytosis was observed by a Zeiss LSM 780 NLO confocal microscope. In addition, the cells were treated with FD@BSA (50, 100, 200 and 400 μg/mL) for 1 h and then stimulated with H_2_O_2_ (250 μM) for 4 h. Cell viability was determined by measuring the absorbance at 450 nm after CCK-8 dye treatment. A mitophagy detection kit (1 μM, 1 h) was used to measure the level of cellular autophagy. The cells were also stained with JC-1 dye to measure the mitochondrial membrane potential (MMP) and treated with an Annexin V-FITC/PI apoptosis kit and a Calcein/PI cytotoxicity assay kit to examine cell viability by confocal microscopy. The probe 2',7'-dichlorodihydrofluorescein diacetate (DCFH-DA; 1 μM, 30 min) was used to measure the level of ROS. The cells were collected, and a flow cytometer and confocal microscope were used to measure the fluorescence intensity. Furthermore, after different treatments, the cells were collected and observed by TEM, and the supernatants were collected and subjected to ELISAs for different inflammatory factors according to the manufacturer's instructions.

### DNA and RNA isolation and real-time qPCR

DNeasy kits (Qiagen) were used to purify cellular DNA. To calculate the mitochondrial DNA (mtDNA)/nuclear DNA (nDNA) ratio, one gene from the mitochondrial genome (D-loop) and one from the nuclear genome (Tert) were amplified by qPCR. Whole-cell RNA was extracted from HK-2 cells using TRIzol reagent. The RNA was reverse transcribed with the Evo M-MLV RT-PCR Kit for cDNA synthesis. The cDNA was then amplified by qPCR with a SYBR Green Premix Pro Taq HS qPCR kit. The primer sequences are listed in Table S1.

### Direct data-independent acquisition (DIA) proteomic analysis

Protein was extracted from cells using SDT lysis buffer (4% SDS, 100 mM DTT, 100 mM Tris-HCl pH 8.0). Cells were boiled for 3 min and further ultra-sonicated. Undissolved cellular debris were removed by centrifugation at 16000 × *g* for 15 min. The supernatant was collected and quantified with a BCA Protein Assay Kit (BeyoTime, China). Briefly, the detergent, DTT and IAA in UA buffer was added to block reduced cysteine. Finally, the protein suspension was digested with trypsin (Promega) at ratio 50:1 overnight at 37 °C. The peptide mixtures were collected by centrifugation at 16000 × *g* for 15 min and desalted with C18 StageTip for further LC-MS analysis. The concentrations of re-dissolved peptides were determined with OD280 by Nanodrop One device (Thermo, USA). LC-MS/MS were performed on a Orbitrap Astral mass spectrometer coupled with Vanquish Neo UHPLC system (Thermo Fisher Scientific). Peptides from each sample were loaded into a column (50 cm Low-Load µPAC™ Neo HPLC Column, Thermo Scientific) at a flow rate of 2.2 μL/min. The RP-HPLC mobile phase A was 0.1% formic acid in water, and B was 0.1% formic acid in 80% acetonitrile. Peptide were eluted over 8 min with a linear gradient of buffer B at 1.25 μL/min. The linear gradient was set as follows: 0-0.1 min, linear gradient from 4% to 6% buffer B; 0.1-1.1 min, linear gradient from 6% to 12% buffer B; 1.1-4.3 min, linear gradient from 12% to 25% buffer B; 4.3-6.1 min, linear gradient from 25% to 45% buffer B; 6.1-6.5 min, linear gradient from 45% to 99% buffer B; 6.5-8 min, buffer B maintained at 99%. The eluted peptides were analyzed on an Orbitrap Astral mass spectrometer. The DIA method consisted of a survey scan from 380-980 m/z at resolution 240000 with AGC target of 500% and 5 ms injection time. The DIA MS/MS scans were acquired by Astral from 150-2000 m/z with 2 m/z isolation window and with AGC target of 500% and 3 ms injection time. Normalized collision energy was set 25 and cycle time was 0.6 s. The spectra of full MS scan and DIA scan were recorded in profile and centroid type respectively. Bioinformatic analysis were carried out with Microsoft Excel and R statistical computing software. Hierarchical clustering analysis and volcano figure were executed with statistical language R. To annotate the sequences, information was extracted from UniProtKB/Swiss-Prot, Kyoto Encyclopedia of Genes and Genomes (KEGG), and Gene Ontology (GO). GO and KEGG enrichment analyses were carried out with the Fisher's exact test, and FDR correction for multiple testing was also performed. GO terms were grouped into three categories: biological process (BP), molecular function (MF), and cellular component (CC). Enriched GO and KEGG pathways were nominally statistically significant at the Fisher exact test p < 0.01. Construction of protein-protein interaction (PPI) networks were also conducted by using the STRING database with the Cytoscape software.

### Western blotting

Cells were collected and processed for protein extraction. A total of 20 μg of each sample was resolved by 10% SDS-PAGE. Afterward, the proteins were transferred onto a 0.22 µm polyvinylidene difluoride (PVDF) membrane (Millipore, USA). The antibodies used for immunoblotting were described in the materials section. A BeyoECL Plus chemiluminescence kit (P0018S, Beyotime) was used to observe the protein bands. Grayscale analysis was performed with ImageJ (version 1.8.0). All the experiments were repeated three times.

### Biosafety and biodistribution analyses

The *in vivo* toxicity of FD@BSA nanozyme was evaluated in mice. All animal experiments were approved by the Ethics Committee of Chongqing Medical University (approval number IACUC-SAHCQMU-2024-00079) with permit number Research Ethics Review No. 223 (2023). Twelve eight-week-old male ICR mice were randomly divided into four groups (n = 3). FD@BSA (100, 200 and 400 μg/mL) was administered by intravenous injection. Mouse blood obtained on day 7 after injection was analyzed on a Sysmex XT-2000i fully automatic hematology analyzer (Kobe, Japan). Moreover, vital organs were obtained and sectioned for hematoxylin and eosin (HE) staining. An Olympus DX51 optical microscope (Tokyo, Japan) was used to observe the pathological changes.

Thirty-six eight-week-old male ICR mice were randomly divided into six groups (n = 3). Cy5-labeled FD@BSA was intravenously administered at a dose of 1 mg/kg. The mice were sacrificed at 0, 2, 4, 6, 12, and 24 h post-injection to obtain the vital organs, including the heart, liver, spleen, lung, and kidney. The distribution of FD@BSA in these organs was visualized with an IVIS Spectrum Imaging System (PerkinElmer, Shanghai, China). Additionally, the major organs were isolated at 2 h for inductively coupled plasma (ICP) analysis. The kidneys were also removed for fluorescence sectioning and biological transmission electron microscopy (bio-TEM) observation (Hitachi, Tokyo, Japan).

### Glycerol-induced AKI mouse model

Twelve eight-week-old male ICR mice were randomly divided into four groups (n = 3). AKI was then induced by 16 h of water deprivation followed by intramuscular injection of 50% glycerol/saline (7 mL/kg). After intramuscular injection, the mice had free access to food and water. The first group received FD@BSA (400 μg/mL) by intravenous injection 4 h after glycerol injection, while another group was treated with NAC as a positive control to assess treatment efficacy. The mice in the control group received no treatment. The therapeutic efficacy of FD@BSA in the AKI model was evaluated by kidney function tests, HE/periodic acid-schiff (PAS) staining and terminal deoxynucleotidyl transferase dUTP nick end labeling (TUNEL) immunofluorescence staining. At 24 h post-injection, the mice were euthanized, and their blood and kidneys were harvested. Blood samples were collected in pediatric heparin tubes and centrifuged at 2000 × *g* for 15 min at 4 ℃. After centrifugation, the blood urea nitrogen (BUN) and creatinine (CRE) levels as well as the levels of different inflammatory factors in the serum were analyzed. For HE/PAS staining analysis, Bax/Bcl-2 immunohistochemical staining and immunofluorescence staining with TUNEL, the collected kidneys were fixed with paraformaldehyde (4% in PBS) and embedded in paraffin, followed by sectioning and staining. After treatment, the kidneys were collected and stored in an optimum cutting temperature specimen matrix for cryostat sectioning at -20 ℃. The frozen kidneys were further sectioned into tissue slices (approximately 5 µm thick). Dihydroethidium (DHE) is a widely used redox-sensitive fluorescent probe which is specific for ROS, such as superoxide and hydrogen peroxide. To assess superoxide production histologically, frozen kidney tissue slices were washed with 1 × PBS and stained with DHE (10 µM) for 30 min. After applying a cover glass to each slide, confocal imaging was performed with a Nikon A1RS confocal microscope (Nikon Instruments). To determine autophagy levels, we sectioned paraffin-embedded renal tissues and performed immunofluorescence staining for the autophagy marker protein LC3 before the samples were photographed and observed with a fluorescence microscope. The autophagic lysosomes in the cells were also observed by bio-TEM (Hitachi, Tokyo, Japan).

### Data analysis

All the experiments were repeated at least three times. Student's unpaired or paired t-tests were used to analyze the significance of the differences between two groups with GraphPad Prism 9.5 software (GraphPad Software, San Diego, CA). We used unpaired multiple t-tests and analysis of variance (ANOVA) to analyze differences among multiple groups. The statistical tests were two-sided, and values of p < 0.05 were considered statistically significant.

## Results and Discussion

### Preparation and characterization of FD@BSA nanozyme

**Figure 1:**
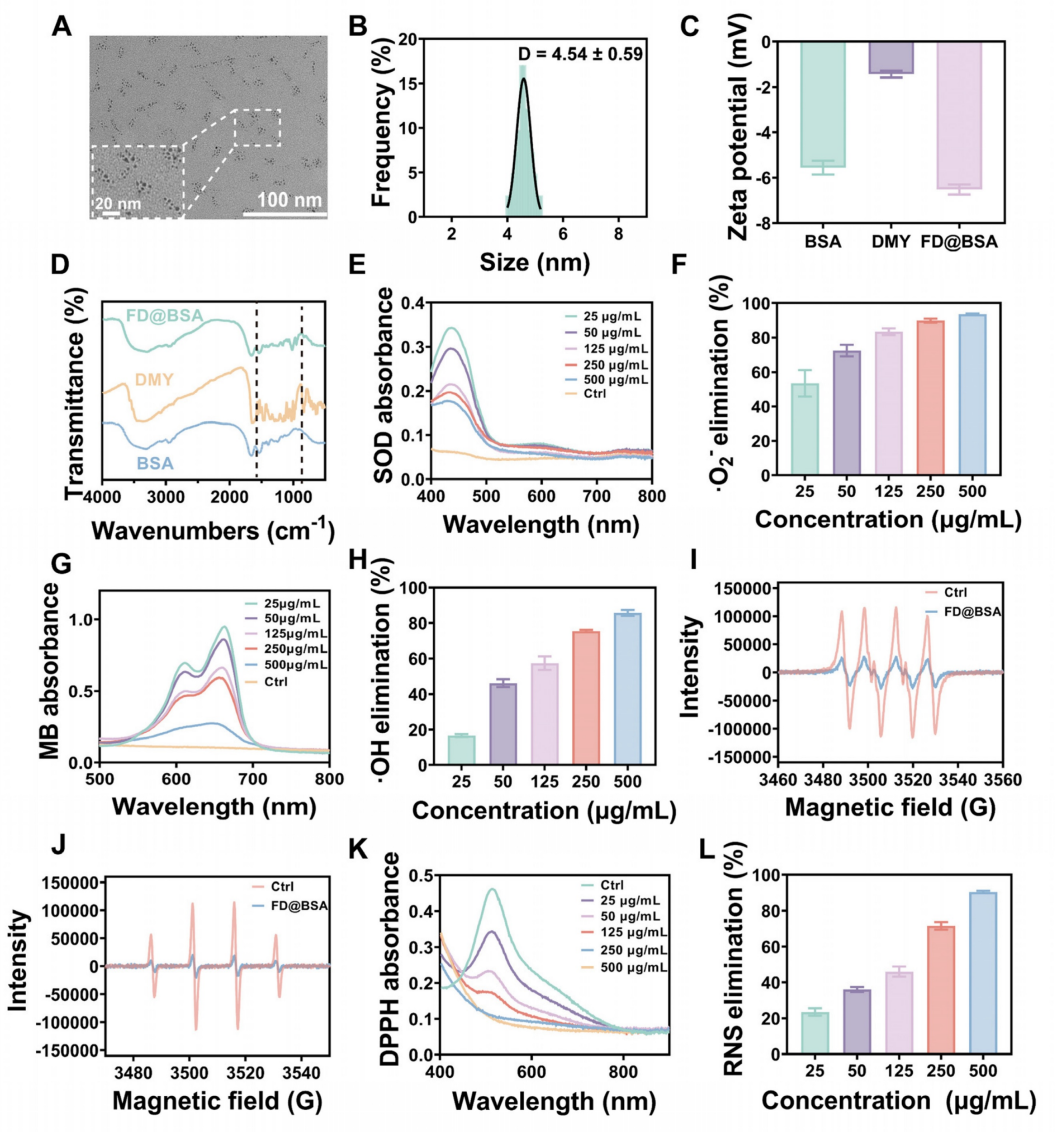
** Characterization of FD@BSA nanozyme.** (A) TEM image of FD@BSA. Scale bar: 100 nm. (B, C, D) Mean hydrodynamic diameter, zeta potential and FT-IR spectrum of FD@BSA. (E, F) SOD-like activity of FD@BSA demonstrated by the WST-8 absorption spectra after reaction with •O_2_^-^ in the presence of various concentrations of FD@BSA (E). FD@BSA concentration-dependent •O_2_^-^ elimination rates (F). (G, H) HORAC of FD@BSA: absorption spectra of MB after reaction with •OH with various concentrations of FD@BSA nanozyme (G). FD@BSA nanozyme concentration-dependent •OH elimination rates (H). (I, J) ESR spectra demonstrating the ability of FD@BSA to eliminate •OH (I) and •O_2_^-^ (J). (K, L) Ability of FD@BSA to eliminate RNS demonstrated by the DPPH absorption spectra after reaction with various concentrations of the FD@BSA nanozyme. (K). FD@BSA concentration-dependent free nitrogen radical elimination rates (L).

Ultrasmall FD@BSA nanozyme was prepared via coordination self‑assembly. BSA was dissolved in ultrapure water, mixed with DMY, and stirred at room temperature for 2 h, followed by addition of FeCl_3_ and overnight stirring. After ultrafiltration and centrifugation, water‑soluble FD@BSA was obtained. TEM image (Figure 1A) revealed uniformly dispersed nanoparticles, and dynamic light scattering showed a hydrodynamic radius of 4.54  ± 0.59 nm with a polydispersity index of 0.225 (Figure 1B). Stability assays over 15 days in ultrapure water and serum demonstrated minimal changes in particle size and polymer dispersity index (PDI) (Figure S1). The zeta potential of -6.3 mV (Figure 1C) indicates the potential for electrostatic interaction with cell membranes. Together, this ultrasmall, negatively charged nanozyme was poised to target and accumulate in the kidney by interacting with positively charged regions of the glomerular filtration barrier and basement membrane 41, 42. FT-IR of FD@BSA revealed a characteristic C-O-C stretching vibration at 950 cm⁻^1^ attributable to DMY, and an amide II band at 1537 cm⁻^1^, confirming BSA incorporation (Figure 1D). Additionally, XPS analysis of the Fe 2p region exhibited peaks at 711.8 and 725.5 eV, corresponding to Fe^3+^ 2p_3/2_ and Fe^3+^ 2p_1/2_, respectively, verifying the trivalent iron state within the nanozyme (Figure S2) 43. In conclusion, FD@BSA was successfully prepared *via* self‑assembly of Fe^3+^ and DMY.

To evaluate the catalytic activity of FD@BSA nanozyme, specifically its superoxide dismutase (SOD)-like activity and hydroxyl radical scavenging capability, we conducted a series of experiments to assess the removal of •OH, •O_2_^-^, and RNS 44. SOD catalyzes the dismutation of •O_2_^-^ to H_2_O_2_, maintaining intracellular redox balance. In our assay, xanthine oxidase-generated •O_2_^-^ was detected using WST-8, which reacts with •O_2_^-^ to form measurable formazan at 450 nm. FD@BSA exhibited dose-dependent •O_2_^-^ scavenging, achieving approximately 85% removal at 125 μg/mL (Figure 1E-F), indicating significant SOD-like activity, which was further quantified in Figure S3. Hydroxyl radical scavenging was assessed *via* a methylene blue discoloration assay, in which •OH neutralization produces a measurable decrease in absorbance at 660 nm. Increasing FD@BSA concentration led to a corresponding reduction in •OH levels (Figure 1G-H). ESR analysis corroborated these findings, showing diminished ESR signal amplitudes for both •O_2_^-^ and •OH in the presence of FD@BSA (Figure 1I-J). Given that H_2_O_2_ is both a major ROS and the product of •O_2_^-^ dismutation, we also evaluated catalase‑like (CAT-like) activity by monitoring O_2_ evolution from H_2_O_2_ decomposition. FD@BSA demonstrated concentration‑dependent H_2_O_2_ scavenging and quantifiable CAT-like kinetics (Figure S4), underscoring its multifunctional antioxidant capabilities.

Moreover, given the pathogenic role of excessive RNS in AKI, we assessed FD@BSA's RNS-scavenging capacity using the stable nitrogen radical DPPH, which contains numerous unpaired electrons (Figure 1K). As shown in Figure 1L, FD@BSA was able to remove 90.5% of DPPH at a concentration of 500 µg/mL. Collectively, FD@BSA nanozyme exhibited powerful ROS/RNS scavenging abilities, demonstrating its ability to remodel the oxidative stress microenvironment.

### Anti-inflammatory effects and repair capabilities of FD@BSA nanozyme *in vitro*

**Figure 2:**
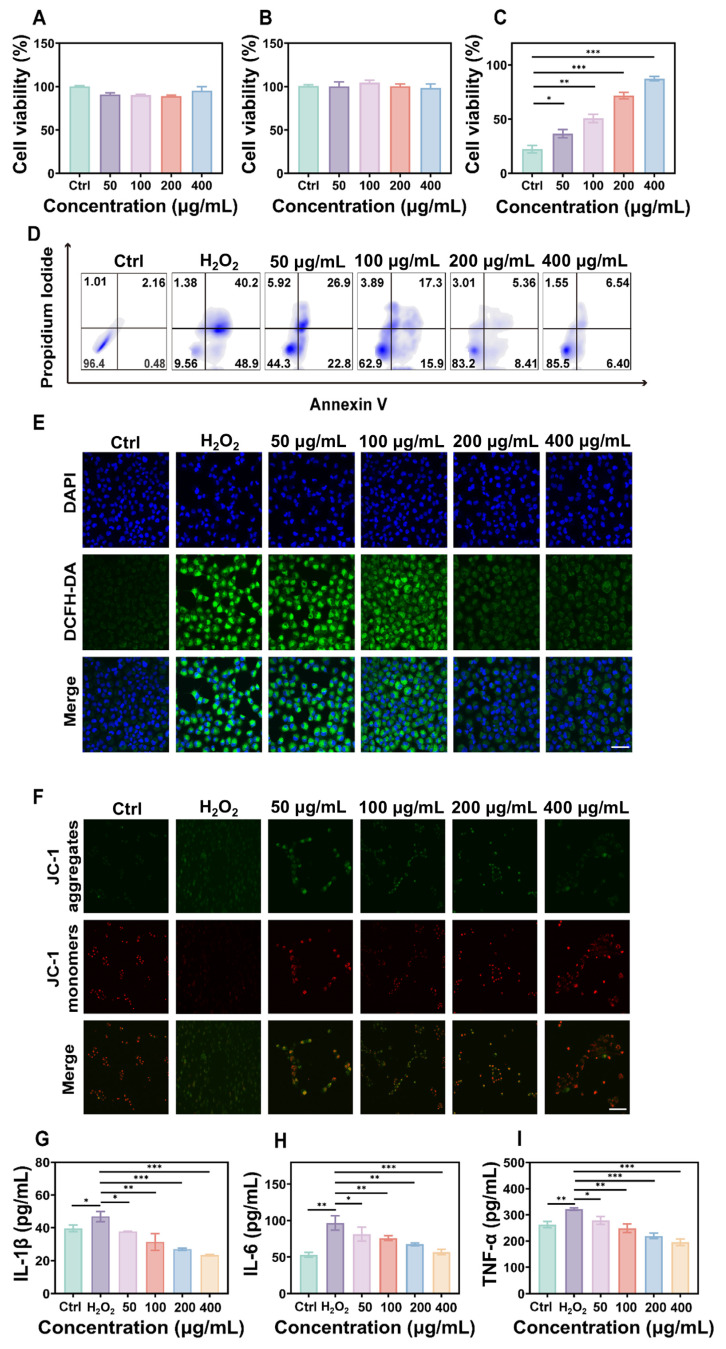
** Anti-inflammatory effects and repair capabilities of FD@BSA nanozyme *in vitro*.** (A, B) Viability of RAW 264.7 and HK-2 cells after coincubation with FD@BSA. The data are presented as the means ± SD (n = 3). (C) Viability of HK-2 cells after stimulation with H_2_O_2_ and treatment with different concentrations of FD@BSA. The data are presented as the means ± SD (n = 3). (D) Apoptotic rate of HK-2 cells after stimulation with H_2_O_2_ and treatment with different concentrations of FD@BSA. (E) CLSM images of ROS in HK-2 cells after stimulation with H_2_O_2_ and treatment with different concentrations of FD@BSA. Scale bar: 50 μm. (F) CLSM images of the changes in the mitochondrial membrane potential after stimulation with H_2_O_2_ and treatment with different concentrations of FD@BSA. Scale bar: 50 μm. (G, H, I) Levels of IL-1β, IL-6 and TNF-α secreted by HK-2 cells after activation by H_2_O_2_ and treatment with different concentrations of FD@BSA. The data are presented as the means ± SD (n = 3). Statistical significance was determined by ANOVA. *p < 0.05, **p < 0.01, ***p < 0.001; ns, not significant.

To evaluate the biocompatibility of FD@BSA nanozyme, we incubated RAW 264.7 cells (mouse macrophages) and HK-2 cells (human renal tubular epithelial cells) with various concentrations of FD@BSA for 24 h. Even at a high concentration of 400 µg/mL, cell viability remained above 85%, indicating minimal cytotoxicity of FD@BSA (Figure 2A-B). Notably, 400 µg/mL FD@BSA significantly improved the viability of HK-2 cells compared with other nanoparticle groups, suggesting a mild pro-proliferative effect of FD@BSA. Moreover, confocal laser scanning microscopy revealed that efficient FD@BSA internalization by HK-2 cells within 6 h (Figure S5).

To further assess the anti-inflammatory and antiapoptotic effects of FD@BSA *in vitro*, we performed a CCK-8 assay to evaluate its protective effects on H_2_O_2_-injured HK-2 cells. As shown in Figure 2C, FD@BSA dose-dependently enhanced survival and reduced cytotoxicity compared with H_2_O_2_ alone. Annexin V-FITC/PI apoptosis analysis revealed that H_2_O_2_ treatment induced 89.1% apoptosis, which decreased to 12.9% following FD@BSA treatment with increasing concentration from 50 to 400 µg/mL (Figure 2D), indicating potent antiapoptotic activity. Additionally, Calcein-AM (green)/PI (red) staining kits confirmed the protective effect of FD@BSA on HK-2 cell growth (Figure S6). To assess ROS scavenging and anti-inflammatory effects, cells were stained with DCFH‑DA and imaged by confocal microscopy. FD@BSA reduced intracellular ROS fluorescence from 80% in the H_2_O_2_ group to 20% (Figure 2E; Figure S7), a finding corroborated by flow cytometry (Figure S8). These results underscore FD@BSA's ability to mitigate oxidative stress-induced injury and remodel the redox microenvironment in renal epithelial cells.

MMP is a sensitive indicator of mitochondrial function, and a decrease of MMP is commonly considered an early marker of cell apoptosis 45. Using JC-1 dye, we evaluated FD@BSA's protection against H_2_O_2_-induced mitochondrial dysfunction. When the cell membrane potential decreases, JC-1 transitions from red fluorescent aggregates to a dispersed state with green fluorescence emission. Confocal laser scanning microscopy showed that H_2_O_2_ treatment markedly increased green fluorescence, whereas FD@BSA dose-dependently restored red aggregate formation, indicating recovery of MMP and mitochondrial function (Figure 2F and Figure S9). When stimulated by various substances, HK-2 cells enter a proinflammatory state and secrete proinflammatory cytokines such as IL-6, TNF-α, and IL-1β, which play crucial roles in the development and progression of AKI. To investigate the anti-inflammatory properties of FD@BSA *in vitro*, we measured the levels of inflammatory cytokines in HK-2 cells. ELISAs confirmed that H_2_O_2_ stimulation significantly elevated these cytokines (Figure 2G-I), consistent with previous studies. In contrast, FD@BSA substantially suppressed the levels of these inflammatory cytokines, demonstrating anti‑inflammatory efficacy. Moreover, FD@BSA may block NLRP3 inflammasome activation by scavenging mitochondrial ROS (mtROS), as mtROS promotes inflammasome assembly *via* mtDNA oxidation and NLRP3 oligomerization 46, 47. JC-1 staining revealed that FD@BSA restored the MMP, further suggesting that FD@BSA interferes with the mtROS-NLRP3 pathway. In summary, FD@BSA remodeled the oxidative stress microenvironment by neutralizing ROS, preserving mitochondrial integrity, and attenuating inflammatory responses, thereby preventing apoptosis.

### Ability of FD@BSA nanozyme to activate autophagy and promote repair *in vitro*

**Figure 3:**
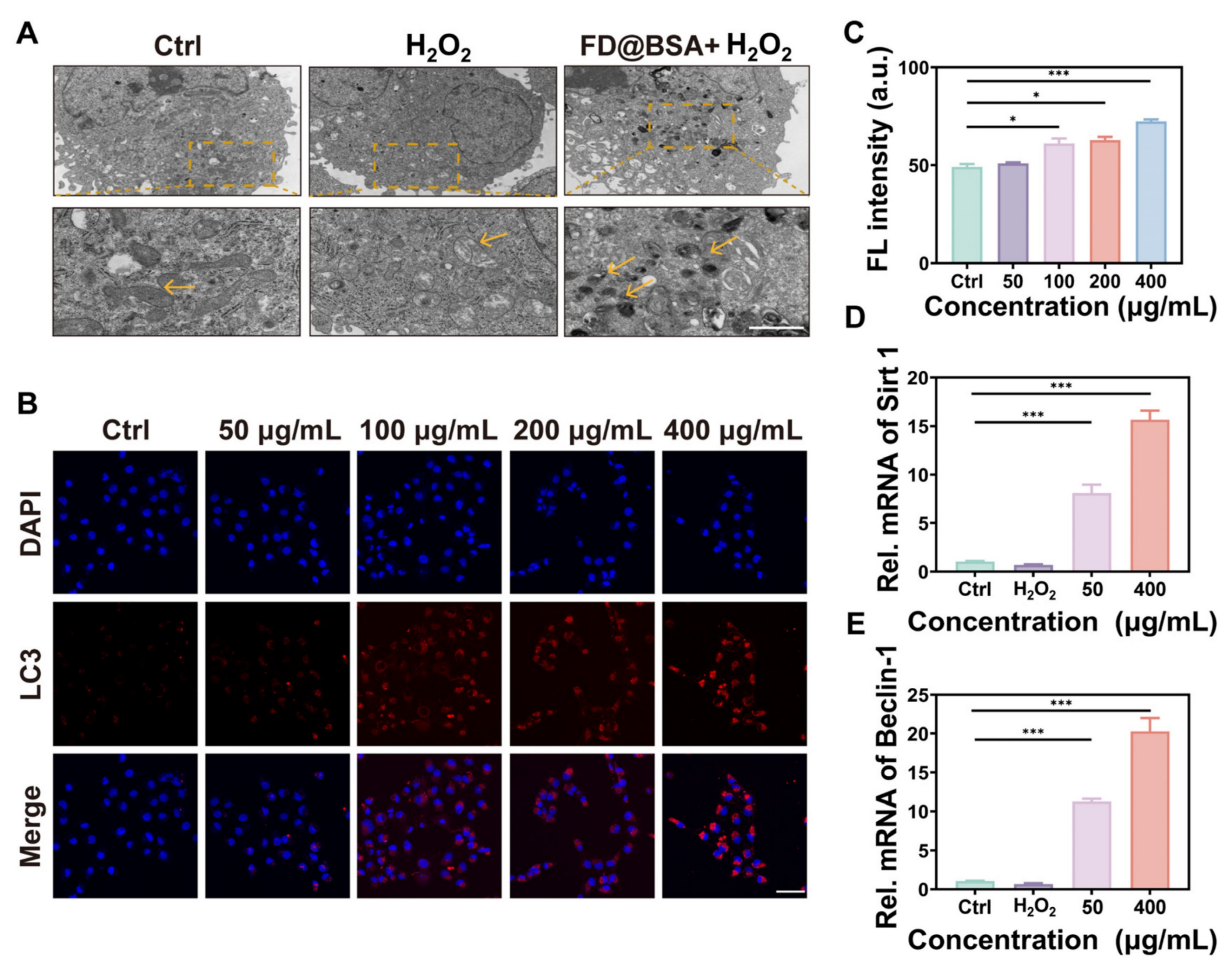
** Autophagy activation *in vitro*.** (A) TEM images of autophagosomes in HK-2 cells after different treatments. Scale bar: 1 μm. (B, C) IF staining of DHE in HK-2 cells after various treatments and quantitative analysis. Scale bar: 50 μm. The data are presented as the means ± SD (n = 3). (D, E) mRNA levels of Beclin-1 and Sirt1 after different treatments. The data are presented as the means ± SD (n = 3). Statistical significance was determined by ANOVA. *p < 0.05, **p < 0.01, ***p < 0.001; ns, not significant.

On the basis of the aforementioned experiments, we hypothesized that FD@BSA could induce mitophagy. To verify this hypothesis, HK-2 cells pre-stimulated with H_2_O_2_ were treated with FD@BSA and prepared as bio-electron microscopy samples. As shown in Figure 3A, untreated cells exhibited normal mitochondria with intact cristae and scant autophagosomes, whereas H_2_O_2_ exposure induced swollen, structurally damaged mitochondria and few autophagosomes. Remarkably, treatment with FD@BSA significantly alleviated these changes, reducing mitochondrial swelling and increasing the number of double-membrane autophagosomes, particularly those surrounding mitochondria (mitophagosomes) and autolysosomes. To confirm these observations, we performed fluorescence staining to assess mitochondrial autophagy, showing a dose-dependent enhancement of mitophagy following FD@BSA exposure (Figure 3B-C). These data indicated that FD@BSA mitigated H_2_O_2_-induced ROS accumulation and promoted cellular repair by synergistically activating autophagy. Mechanistically, FD@BSA scavenged excess ROS to alleviate inhibition of lysosomal acidification and upregulates Sirt1, facilitating Beclin-1 deacetylation and autophagosome biogenesis 48, 49.

Furthermore, we examined whether FD@BSA promoted autophagy *via* the Sirt1 pathway. qPCR analysis revealed that FD@BSA markedly upregulated Sirt1 and Beclin-1 mRNA levels, which are the key autophagy markers (Figure 3D-E), corroborating FD@BSA's activation of mitophagy *via* the Sirt1-Beclin-1 axis.

### Proteomic analysis and pathway validation

**Figure 4:**
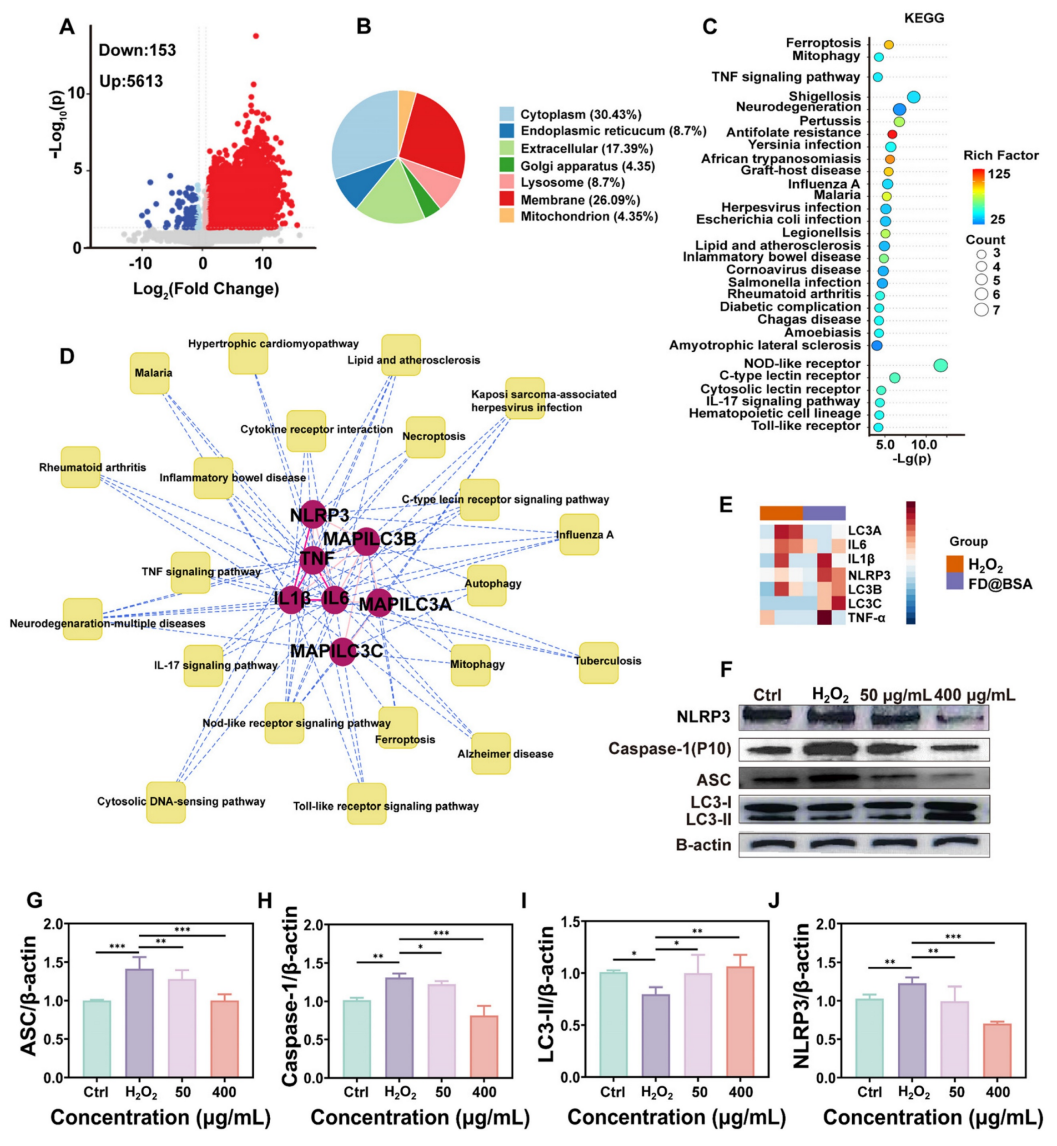
** Proteomic analysis and pathway validation.** (A) Volcano plot of the differentially expressed proteins in HK-2 cells after stimulation with H_2_O_2_ and treatment with FD@BSA. (B) Localization analysis of the differentially expressed proteins in HK-2 cells after stimulation with H_2_O_2_ and treatment with FD@BSA. (C) Pathway enrichment analysis. (D) The most significant module, which included 7 hub genes. (E) Heatmap. (F, G, H, I, J) WB analysis of the expression of key proteins involved in mitochondrial autophagy and several inflammation-related signaling pathways and the corresponding quantitative analysis. The data are presented as the means ± SD (n = 3). Statistical significance was determined by ANOVA. *p < 0.05, **p < 0.01, ***p < 0.001; ns, not significant.

To further investigate the anti-inflammatory and autophagy activation effects of the nanozyme, we conducted proteomic analyses of the H_2_O_2_-injured and FD@BSA-treated groups. The volcano plot revealed that approximately 200 proteins were downregulated, whereas approximately 6000 proteins were upregulated (Figure 4A). Subcellular localization analysis indicated predominant cytoplasmic distribution, with marked enrichment of lysosomal and mitochondrial autophagy-related proteins (Figure 4B). KEGG enrichment of differentially expressed proteins highlighted the top 28 pathways, notably mitophagy and inflammation-associated signaling cascades (Figure 4C). Significant enrichment within the autophagy-lysosome pathway suggests that FD@BSA may enhance autophagic flux by restoring lysosomal function (e.g., enhancing histone activity) 21. Intersection analysis of enriched pathways underscored FD@BSA's central regulatory role in oxidative stress models (Figure 4D). Finally, protein-protein interaction mapping identified IL-6, the NLRP3 inflammasome, TNF-α, and key autophagy regulators as hubs mediating the shift toward an anti-inflammatory, pro‑autophagic microenvironment that facilitated cellular repair by FD@BSA treatment (Figure 4E).

Additionally, Western blot analysis demonstrated that FD@BSA treatment inhibited activation of the NLRP3 inflammasome in HK-2 cells. FD@BSA treatment also suppressed Caspase-1 release and the formation of ASC protein “specks” in the perinuclear region, which are large spherical structures formed by self-association prior to downstream signaling activation upon H_2_O_2_ stimulation. Western blotting also revealed a significant increase in the protein expression of the autophagy marker LC3, indicating the formation of mitophagosomes and mitolysosomes (Figure 4F). During autophagy, cytosolic LC3-I undergoes lipidation to form LC3-II, which associates with autophagosome membranes and serves as a quantitative marker of autophagic flux 50-52. Grayscale analysis of LC3-II bands confirmed a significant increase in autophagic activity following FD@BSA treatment (Figure 4G-J).

In conclusion, FD@BSA exhibited potent antioxidant and anti-inflammatory activities by inhibiting inflammation-related protein expression. It effectively remodeled the intracellular oxidative stress environment to alleviate oxidative damage and upregulated Sirt1 and Beclin-1 in HK-2 cells, activating mitophagy and facilitating cellular repair.

### Biosafety and biodistribution of FD@BSA nanozyme *in vivo*

**Figure 5:**
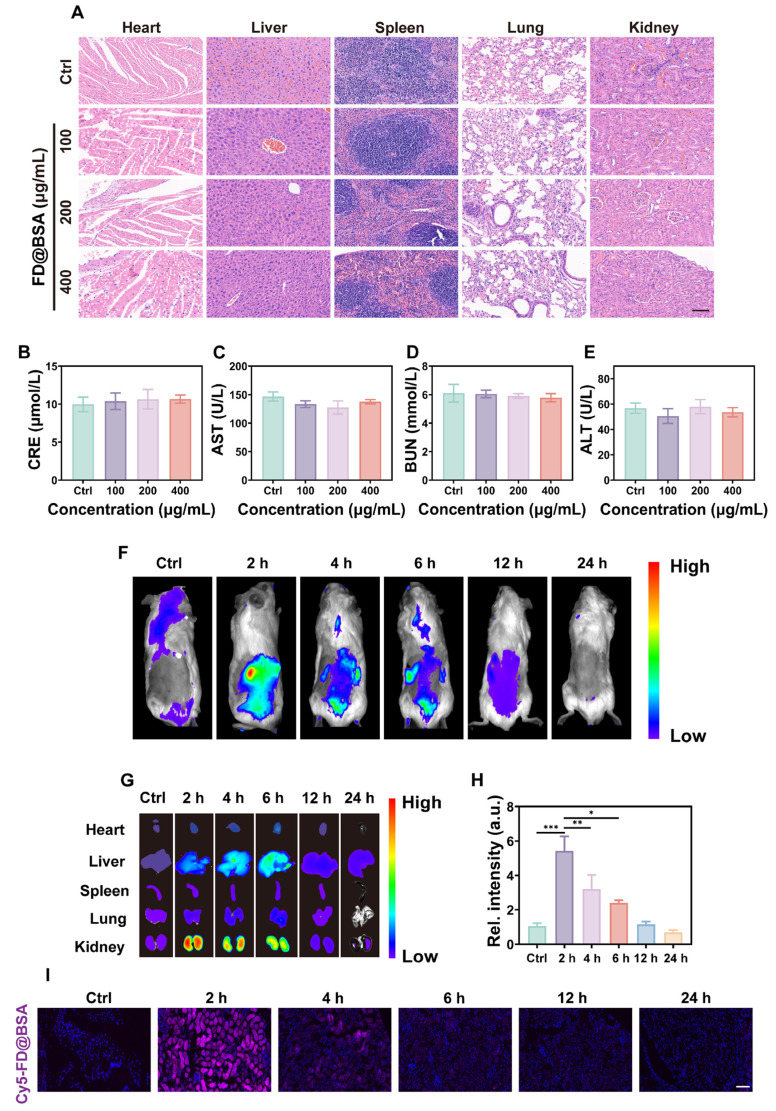
** Biosafety and biodistribution of FD@BSA nanozyme *in vivo*.** (A) HE staining images of major organs treated with different concentrations of FD@BSA. Scale bar: 50 μm. (B-E) Blood biochemical parameters of mice after intravenous injection of FD@BSA. The data are presented as the means ± SD (n = 3). (F) *In vivo* FL images of mice captured at different time points after injection of Cy5-labeled FD@BSA. (G, H) The distribution of FD@BSA in *ex vivo* FL images of major organs (heart, liver, spleen, lung, and kidney) before injection and at different time points after and quantitative analysis of FL intensity in the kidney. (I) Cy5-labeled FD@BSA in kidney sections. Scale bar: 100 μm. The data are presented as the means ± SD (n = 3). Statistical significance was determined by ANOVA. *p < 0.05, **p < 0.01, ***p < 0.001; ns, not significant.

The biosafety of FD@BSA nanozyme was evaluated *in vivo via* intravenous administration (100, 200, or 400 μg/mL). Blood samples and major organs (heart, liver, spleen, lung, and kidney) were collected 24 h post-injection. Histopathology showed no renal damage or alterations in other organs, even at the highest dose (Figure 5A), indicating negligible organ toxicity. Serum biomarkers of kidney and liver function and hematological parameters remained within normal limits (Figure 5B-E). Hemolysis assays using mouse erythrocytes revealed no hemolytic activity across the tested concentrations (Figure S10). Moreover, the survival rates did not differ from controls. These results demonstrated the excellent *in vivo* biocompatibility and low toxicity of FD@BSA, supporting its potential for clinical application.

To further investigate the biodistribution of FD@BSA, *in vivo* fluorescence imaging of Cy5-labeled FD@BSA was conducted. As shown in Figure 5F, the abdominal fluorescence intensity peaked at 2 h post-injection and decreased markedly by 24 h, indicating rapid nanoparticle clearance. *Ex vivo* imaging of organs at different time points revealed that preferential accumulation in the liver and kidney, with only faint fluorescence in the heart, spleen, and lung. Notably, the fluorescence intensities in the kidney were 5.4- and 3.1-fold higher than hepatic signal at 2  and 4 h, respectively (Figure 5G-H). In addition, Confocal imaging of kidney sections confirmed these distributions described above (Figure 5I). After 24 h, almost no fluorescence was detected in the *ex vivo* organs, demonstrating efficient metabolism of FD@BSA. Biological TEM images of renal tissues revealed initial FD@BSA localization within glomerular capillaries at 0-2 h, indicating size-selective filtration. At 2-4 h, intracellular accumulation within lysosome-rich regions of tubular epithelial cells suggested lysosomal degradation common to protein carriers. By 6-12 h, glomerular and tubular nanoparticle levels had significantly declined, indicating rapid urinary excretion (Figure S11). This corresponds to the reported 6 h renal clearance of sub-10 nm nanoparticles 53. Finally, we quantified the metabolism of FD@BSA, and the results revealed a half-life of 2.33 h (Figure S12), as determined by ICP analysis of Fe content in liver and kidney (Figure S13). These data suggested that the ultrasmall hydrodynamic diameter (~4.54 nm) of FD@BSA enabled efficient renal passage and kidney targeting within the ~6 nm filtration threshold 54. Collectively, FD@BSA exhibited favorable biodistribution, rapid clearance, and excellent *in vivo* biosafety.

### Evaluation of therapeutic effects in AKI mice

**Figure 6:**
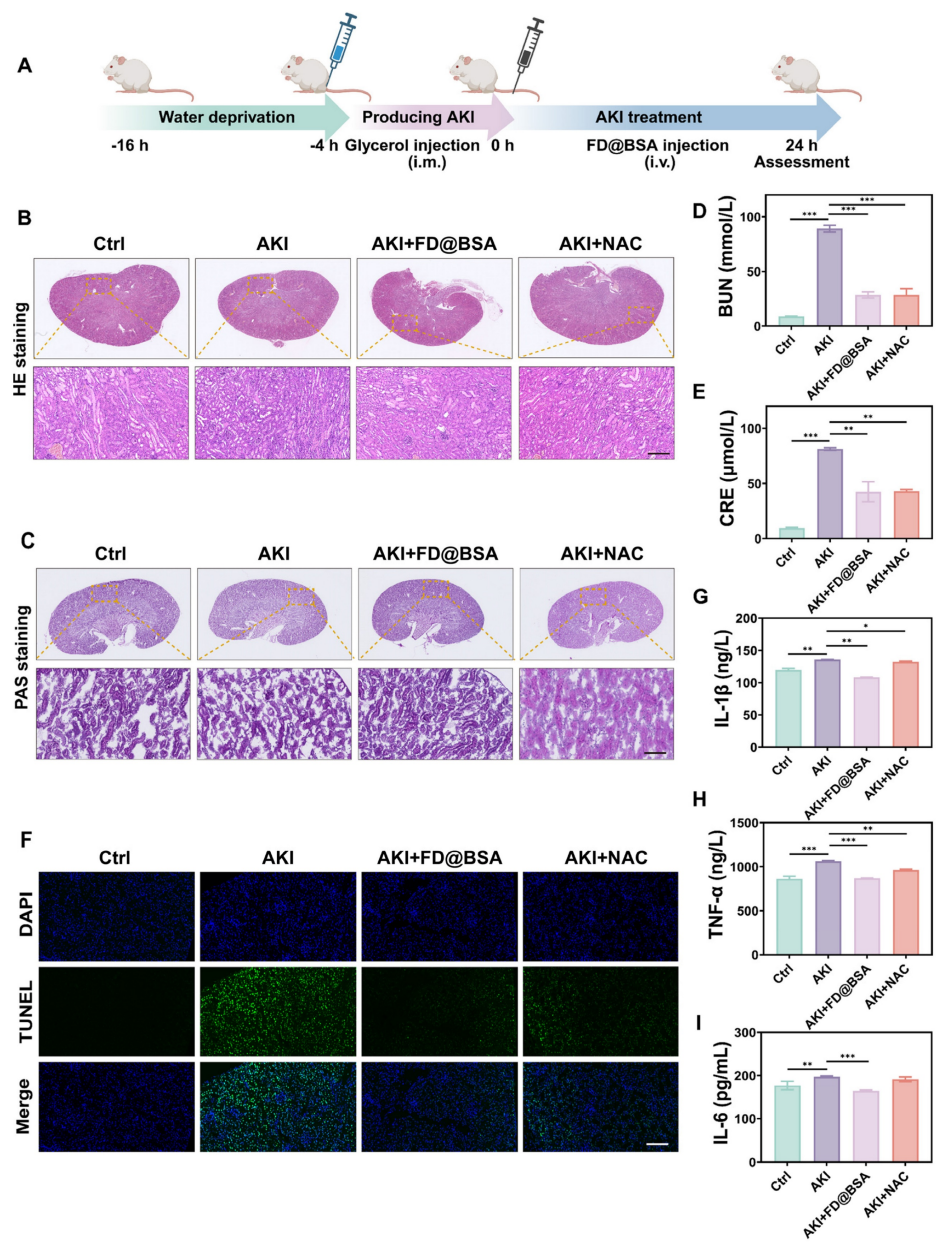
** Therapeutic effects of FD@BSA *in vivo*.** (A) Schematic of the experimental procedure for exploring the therapeutic effects of FD@BSA in AKI mice. (B) HE staining images of kidney tissues. Scale bar: 100 µm. (C) PAS-stained kidney tissues. Scale bar: 100 µm. (D, E) Serum levels of BUN and CRE after different treatments. (F) Representative TUNEL staining images of kidney slices after different treatments. Scale bar: 50 μm. (G, H, I) Levels of IL-1β, IL-6 and TNF-α in HK-2 cells after different treatments. The data are presented as the means ± SD (n = 3). Statistical significance was determined by ANOVA. *p < 0.05, **p < 0.01, ***p < 0.001; ns, not significant.

The glycerol-induced AKI model was used to evaluate the therapeutic efficacy of FD@BSA as a potential treatment. The model was established by water deprivation for 16 h to induce rhabdomyolysis and hemolysis, followed by intramuscular injection of 50% glycerol/saline to provoke oxidative stress-related kidney damage and subsequent renal dysfunction. 4 h after model induction, mice received FD@BSA (10 mL/kg, *i.v.*) or NAC (7 mL/kg,* i.v.*) as a positive control (Figure 6A). Histological evaluation (HE and PAS staining) revealed that FD@BSA markedly attenuated tubular necrosis, cast formation, and epithelial detachment in AKI kidneys in AKI mice (Figure 6B-C). Consistent with these findings, serum creatinine and blood urea nitrogen levels were significantly reduced in FD@BSA-treated mice compared with untreated AKI controls (Figure 6D-E). TUNEL staining further confirmed a substantial decrease in renal cell apoptosis following FD@BSA administration (Figure 6F), demonstrating its therapeutic efficacy in mitigating AKI.

To investigate the *in vivo* antioxidant capacity of FD@BSA and its impact on inflammatory cytokines, the serum levels of IL-1β, IL-6, and TNF-α were measured using ELISA. As expected, AKI control mice displayed marked elevations in all three cytokines, whereas FD@BSA treatment significantly reduced IL-1β, IL-6, and TNF-α levels (Figure 6G-I). These findings suggested that FD@BSA effectively attenuated oxidative stress and inflammatory responses, thereby facilitating renal repair, showing great promise as a therapeutic agent for AKI.

### Anti-inflammatory and autophagy activation mechanisms *in vivo*

**Figure 7:**
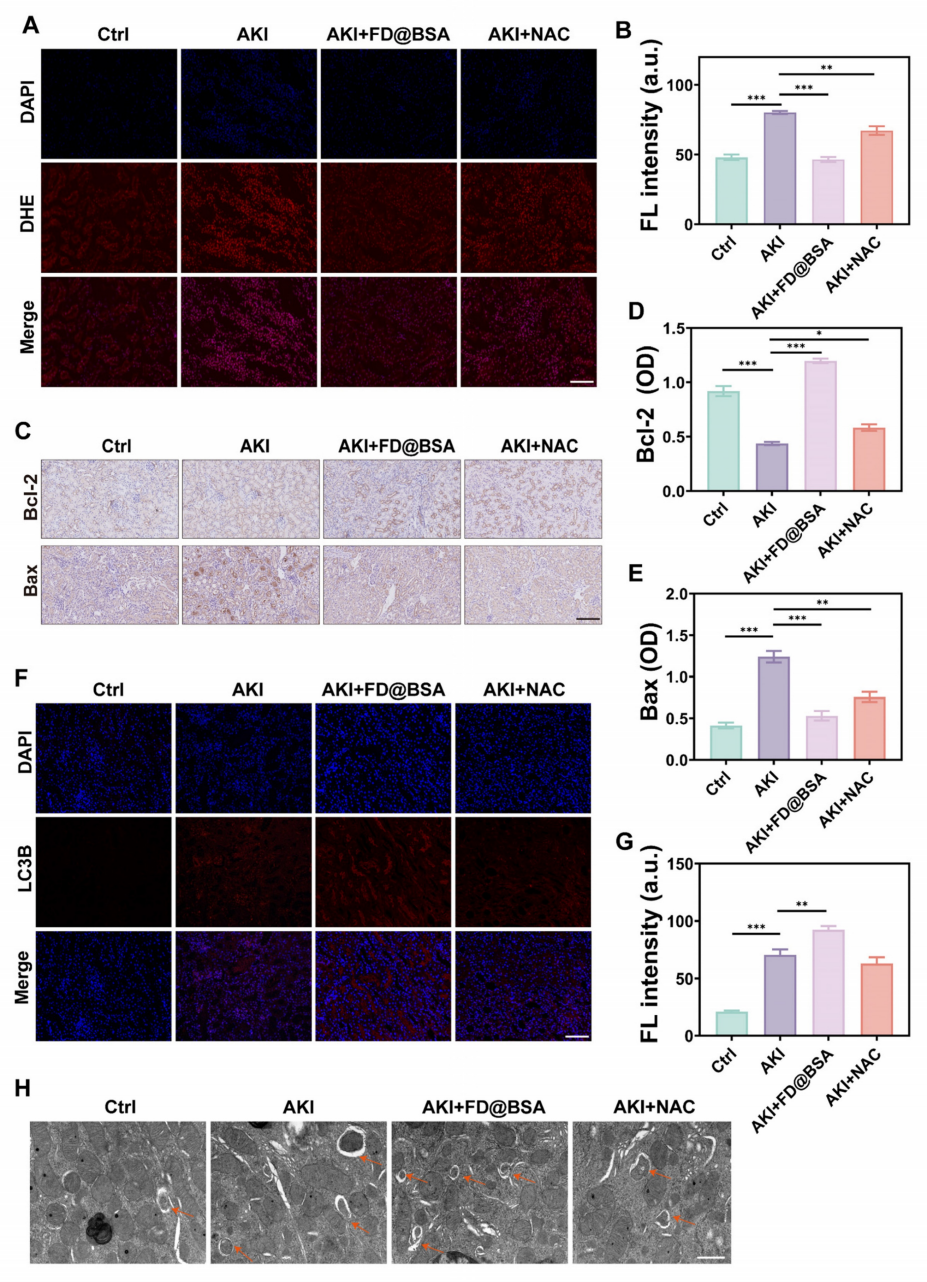
** Anti-inflammatory and autophagy activation mechanisms *in vivo*.** (A, B) IF staining of DHE in kidney sections after various treatments (scale bar: 50 μm) and the corresponding quantitative analysis. The data are presented as the means ± SD (n = 3). (C-E) Immunohistochemical staining of Bax and Bcl-2 in kidney sections after various treatments (scale bar: 100 μm) and the corresponding quantitative analysis. The data are presented as the means ± SD (n = 3). (F, G) IF staining of LC3 in kidney sections after various treatments (scale bar: 50 μm) and the corresponding quantitative analysis. (H) TEM was performed to identify autolysosomes in the kidney after different treatments. Scale bar: 1 μm. The data are presented as the means ± SD (n = 3). Statistical significance was determined by ANOVA. *p < 0.05, **p < 0.01, ***p < 0.001; ns, not significant.

Encouraged by its *in vitro* antioxidant capacity of the nanozyme, we further investigated whether FD@BSA could effectively scavenge ROS, particularly superoxides, generated in the damaged kidneys of AKI mice. As expected, glycerol-induced kidney cells generated large amounts of superoxide, resulting in strong red fluorescence signals in DHE-stained sections from the AKI model group (Figure 7A). In contrast, FD@BSA treatment reduced DHE fluorescence by ~50%, restoring signal intensity nearly to that of healthy controls, whereas NAC achieved only a 28.7% reduction (Figure 7B). These results demonstrated that FD@BSA more effectively eliminated ROS in AKI kidneys than NAC.

Bax is a proapoptotic protein located primarily on the outer mitochondrial membrane. As a single-stranded membrane protein, Bax plays a crucial role in regulating apoptosis. Bax activation and aggregation lead to changes in mitochondrial membrane permeability, causing the release of apoptotic molecules such as cytochrome c. Conversely, Bcl-2 is an antiapoptotic protein that is found mainly on the outer mitochondrial membrane and other organelles, such as the endoplasmic reticulum. Bcl-2 primarily inhibits Bax activity, preventing changes in mitochondrial membrane permeability and apoptosis. The Bcl-2/Bax ratio therefore serves as an indicator of cellular anti‑apoptotic capacity and survival. Immunohistochemistry of kidney sections demonstrated marked upregulation of Bax and downregulation of Bcl-2 in glycerol-induced AKI mice versus healthy controls. FD@BSA treatment reversed these changes in a dose-dependent manner, reducing Bax expression and elevating Bcl-2 levels, thereby confirming its potent antiapoptotic effect (Figure 7C-E).

Furthermore, the expression of LC-3, a classic autophagy marker, was analyzed through immunofluorescence staining (Figure 7F). Compared with NAC, FD@BSA not only exhibited superior ROS scavenging in the kidneys but also enhanced autophagy, promoted mitophagy, improved mitochondrial function, and maintained intracellular homeostasis (Figure 7G). Bio-TEM further revealed increased autolysosome formation in FD@BSA-treated group (Figure 7H). These data indicated that FD@BSA exerted its therapeutic efficacy primarily through ROS neutralization, suppression of inflammatory signaling, modulation of the oxidative stress microenvironment, and activation of autophagic pathways, thereby attenuating AKI and promoting renal recovery.

## Conclusion

In summary, we successfully engineered an ultrasmall FD@BSA nanozyme exhibiting robust SOD and CAT-like activities as well as hydroxyl radical scavenging capacity. FD@BSA efficiently scavenged reactive oxygen and nitrogen species (RONS), conferred cytoprotection *in vitro* by enhancing autophagy and attenuating inflammatory signaling, and exhibited rapid renal accumulation, high clearance, and excellent biocompatibility *in vivo*. Therapeutically, FD@BSA improved renal function, mitigated tubular injury, and elevated autophagic flux. Mechanistic studies revealed that FD@BSA suppressed inflammation, remodeled the oxidative stress microenvironment, and activated mitophagy to promote cellular repair. Together, these findings established FD@BSA as a multifunctional nanoplatform for anti-inflammatory therapy in AKI and other oxidative stress-related diseases.

## Supplementary Material

Supplementary figures and table.

## Abbreviations

AKI: acute kidney injury
ASC: apoptosis-associated speck-like protein containing a CARD
Bax: Bcl-2associated X protein
Bcl-2: B-cell lymphoma-2
BSA: bovine serum albumin
BUN: blood urea nitrogen
CAT: catalase
CCK-8: cell counting kit-8
CRE: creatinine
DCFH-DA: 2',7'-dichlorodihydrofluorescein diacetate
DHE: dihydroethidium
DMY: dihydromyricetin
ELISA: enzyme-linkedimmunosorbent assay
ESR: electron spin resonance
FBS: fetal bovine serum
FT-IR: fourier transform infrared spectroscopy
HE: hematoxylin and eosin
HORAC: hydroxyl radical antioxidant capacity
ICP: inductively coupled plasma
LC3: light chain 3
MAPK: mitogen-activated protein kinase
MMP: mitochondrial membrane potential
mtROS: mitochondrial ROS
NAC: N-acetylcysteine
NLRP3: NOD-like receptor family pyrin domain containing 3
PAS: periodic acid-schiff
PDI: polymer dispersity index
PI3K: phosphoinositide 3-kinase
RNS: reactive nitrogen species
ROS: reactive oxygen species
Sirt1: Sirtuin 1
SOD: superoxide dismutase
TEM: transmission electron microscopy
TUNEL: terminal deoxynucleotidyl transferase dUTP nick end labeling
XPS: X-ray photoelectron spectroscopy
