# Supplementary material for: Fe-flavonoid nanozyme as dual modulator of oxidative stress and autophagy for acute kidney injury repair
Source: Theranostics. 2025 Jul 28;15(16):8658–74. doi: 10.7150/thno.111874 (PMC12374728; doi:10.7150/thno.111874)
Supplement: Supplementary file 1 — Supplementary figures and table. [file thnov15p8658s1.pdf]

Supporting information

Fe-flavonoid nanozyme as dual modulator of oxidative stress and autophagy for acute kidney injury repair

Ranran Luo[1, #], Zhongsheng Xu[1, #], Chenxi Zhang[1], Zening Zhang[1], Pengchen Ren[1], Xiaojing He[1, *], Jingjing Zhang[2-5, *], Yun Liu[1, *]

[1]*Department of Radiology, the Second Affiliated Hospital of Chongqing Medical University, Chongqing, 400010, P. R. China.*

[2]*Departments of Diagnostic Radiology, Surgery, Chemical and Biomolecular Engineering, and Biomedical Engineering, Yong Loo Lin School of Medicine and College of Design and Engineering, National University of Singapore, Singapore 119074, Singapore.*

[3]*Clinical Imaging Research Centre, Centre for Translational Medicine, Yong Loo Lin School of Medicine, National University of Singapore, Singapore 117599, Singapore.*

[4]*Nanomedicine Translational Research Program, Yong Loo Lin School of Medicine, National University of Singapore, Singapore 117597, Singapore.*

[5]*Theranostics Center of Excellence (TCE), Yong Loo Lin School of Medicine, National University of Singapore, 11 Biopolis Way, Helios, Singapore 138667, Singapore.*

[#]*These authors contributed equally to this work.*

**Corresponding author: yunliu@cqmu.edu.cn (Yun Liu,), he_xiaojing@hospital.cqmu.edu.cn (Xiaojing He), and j.zhang@nus.edu.sg (Jingjing Zhang).*

Gene	Forward	Reverse
Sirt 1	GAGGAGCGGCAGGAGTATG	CAGGAACTTGGGGTTGACATC
Beclin-1	AGATGCGCTATGCCCAGATG	AAGCGACCCAGTCTGAAATTATTGA
GADPH	GGCACAGTCAAGGCTGAGAATG	ATGGTGGTGAAGACGCCAGTA

Table S1. Primers used in this study

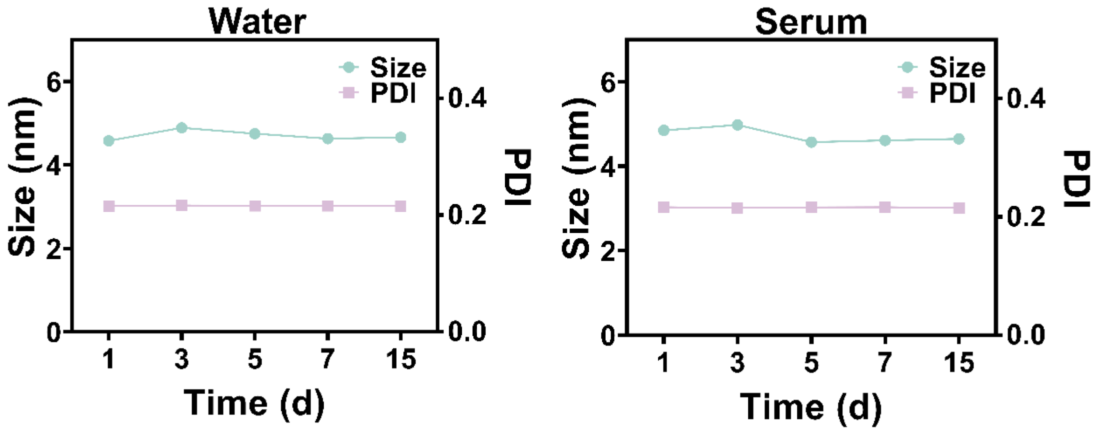

Figure S1. Hydrated particle size and PDI changes in 15 days.

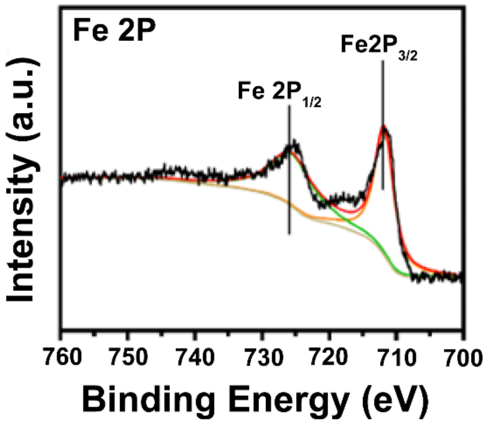

Figure S2. XPS spectrum of Fe 2P.

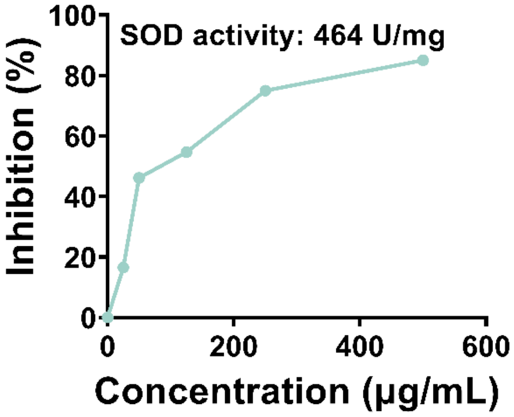

Figure S3. Inhibition rate increased as the higher SOD-like activity of FD@BSA.

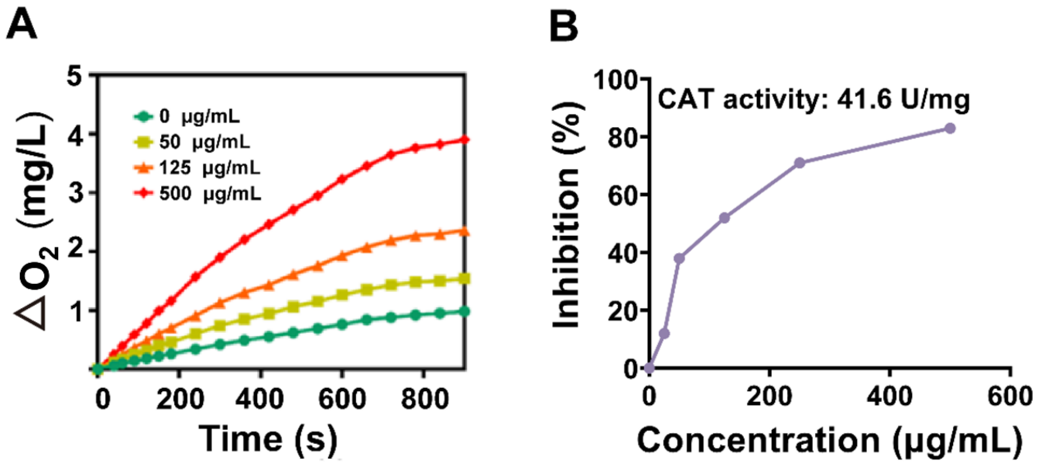

Figure S4. (A)The CAT-like ability of FD@BSA and (B) corresponding enzyme activity calculation.

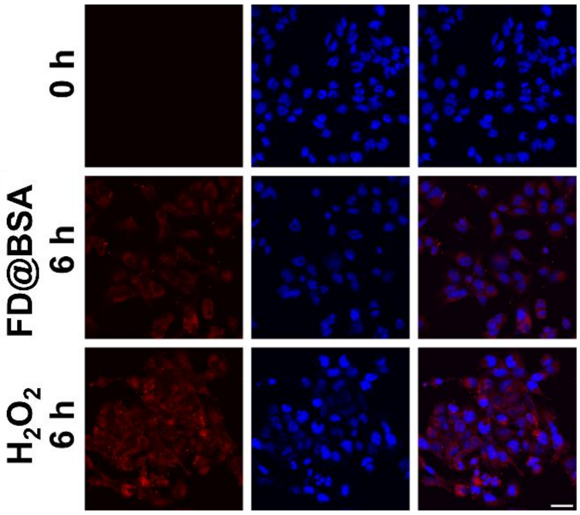

Figure S5. CLSM images of intracellular uptake of Cy5-labeled FD@BSA and H_2O_2 after incubation with HK-2 cells. Scale bar: 50 μm.

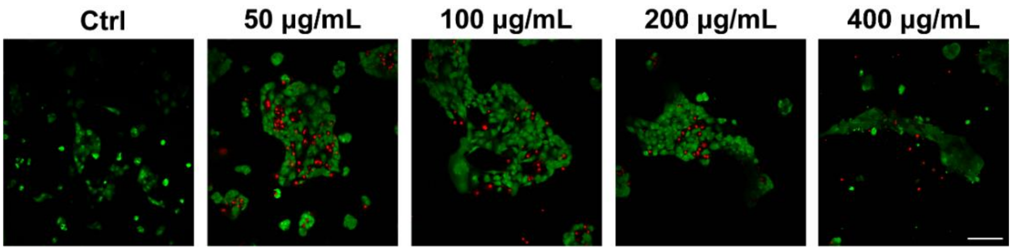

Figure S6. Live/dead cells staining of HK-2 cells after being stimulated with H_2O_2 and treatment with different amounts of FD@BSA under a FL microscope. Scale bar: 50 μm.

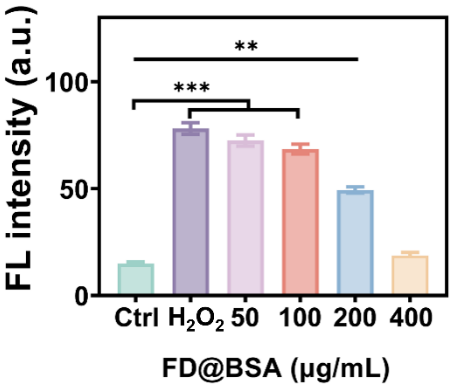

Figure S7. FL intensity of ROS. Data are shown as the mean values ± SD (n = 3). All the statistical significance was analyzed by ANOVA. * $p < 0.05$, ** $p < 0.01$, *** $p < 0.001$, ns, not significant.

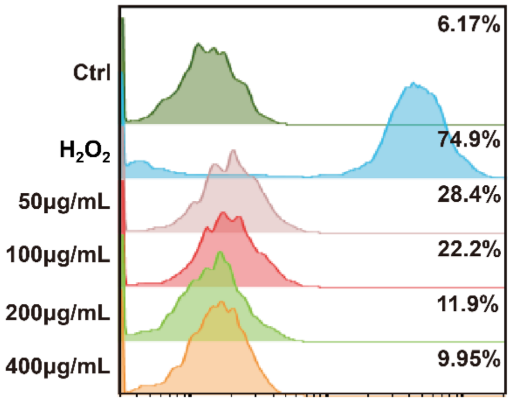

Figure S8. FCM of ROS analysis

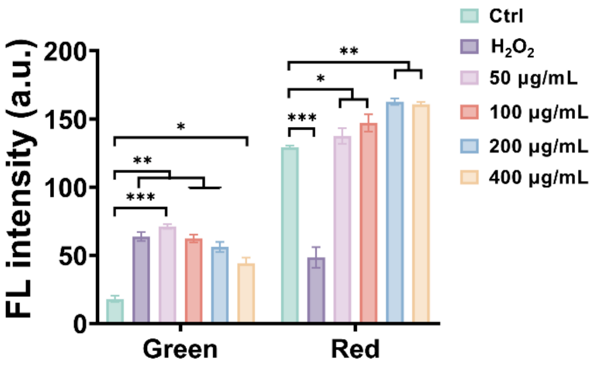

Figure S9. FL intensity of JC-1 monomers and aggregates. Data are shown as the mean values ± SD (n = 3). All the statistical significance was analyzed by ANOVA. * $p < 0.05$, ** $p < 0.01$, *** $p < 0.001$, ns, not significant.

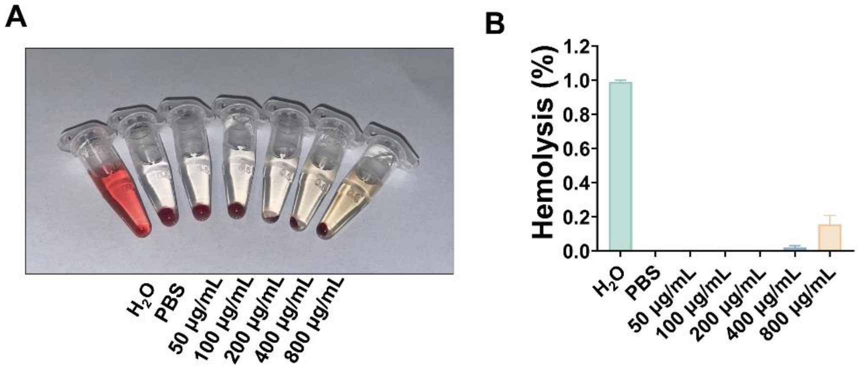

Figure S10. The representative digital photos of hemolysis tests using mouse red blood cells (A) and the corresponding quantitative analysis (B).

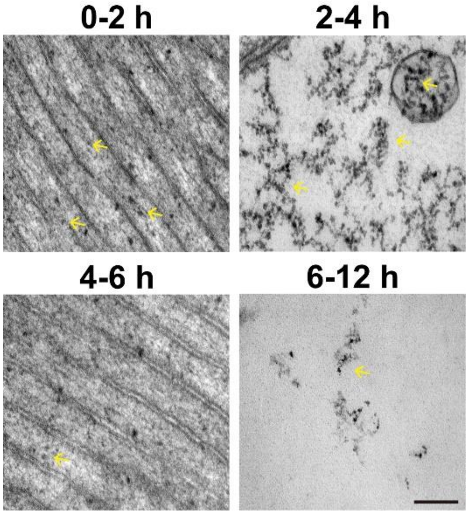

Figure S11: TEM of mouse kidney at different time intervals. Scale bar : 200 nm

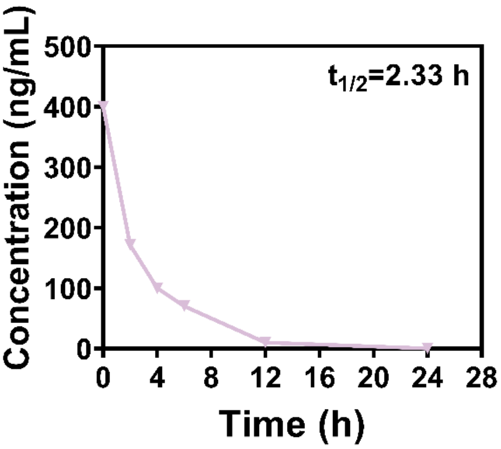

Figure S12. Half-life of FD@BSA metabolism in mice.

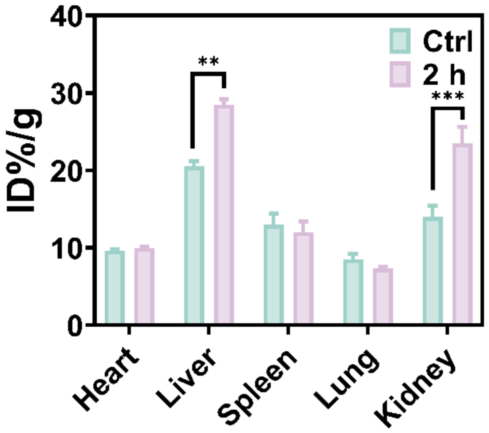

Figure S13. Biodistribution of FD@BSA in mice at 2 h post-injection. All the statistical significance was analyzed by ANOVA. * $p < 0.05$, ** $p < 0.01$, *** $p < 0.001$, ns, not significant.
